# Time course of cerebral oxygenation and cerebrovascular reactivity in Kyrgyz highlanders. A five-year prospective cohort study

**DOI:** 10.3389/fphys.2023.1160050

**Published:** 2023-10-10

**Authors:** Matthias C. Luyken, Paula Appenzeller, Philipp M. Scheiwiller, Mona Lichtblau, Maamed Mademilov, Aybermet Muratbekova, Ulan Sheraliev, Ainura Abdraeva, Nuriddin Marazhapov, Talant M. Sooronbaev, Silvia Ulrich, Konrad E. Bloch, Michael Furian

**Affiliations:** ^1^ Department of Respiratory Medicine, University Hospital Zurich, University of Zurich, Zurich, Switzerland; ^2^ Swiss-Kyrgyz High-Altitude Medicine and Research Initiative, Bishkek, Kyrgyzstan; ^3^ National Centre for Cardiology and Internal Medicine, Bishkek, Kyrgyzstan

**Keywords:** altitude, highlanders, near-infrared spectroscopy, cerebral oxygenation, hyperoxia, hypobaric hypoxia, hypocapnia

## Abstract

**Introduction:** This prospective cohort study assessed the effects of chronic hypoxaemia due to high-altitude residency on the cerebral tissue oxygenation (CTO) and cerebrovascular reactivity.

**Methods:** Highlanders, born, raised, and currently living above 2,500 m, without cardiopulmonary disease, participated in a prospective cohort study from 2012 until 2017. The measurements were performed at 3,250 m. After 20 min of rest in supine position while breathing ambient air (FiO_2_ 0.21) or oxygen (FiO_2_ 1.0) in random order, guided hyperventilation followed under the corresponding gas mixture. Finger pulse oximetry (SpO_2_) and cerebral near-infrared spectroscopy assessing CTO and change in cerebral haemoglobin concentration (cHb), a surrogate of cerebral blood volume changes and cerebrovascular reactivity, were applied. Arterial blood gases were obtained during ambient air breathing.

**Results:** Fifty three highlanders, aged 50 ± 2 years, participated in 2017 and 2012. While breathing air in 2017 vs. 2012, *P*aO_2_ was reduced, mean ± SE, 7.40 ± 0.13 vs. 7.84 ± 0.13 kPa; heart rate was increased 77 ± 1 vs. 70 ± 1 bpm (*p* < 0.05) but CTO remained unchanged, 67.2% ± 0.7% vs. 67.4% ± 0.7%. With oxygen, SpO_2_ and CTO increased similarly in 2017 and 2012, by a mean (95% CI) of 8.3% (7.5–9.1) vs. 8.5% (7.7–9.3) in SpO_2_, and 5.5% (4.1–7.0) vs. 4.5% (3.0–6.0) in CTO, respectively. Hyperventilation resulted in less reduction of cHb in 2017 vs. 2012, mean difference (95% CI) in change with air 2.0 U/L (0.3–3.6); with oxygen, 2.1 U/L (0.5–3.7).

**Conclusion:** Within 5 years, CTO in highlanders was preserved despite a decreased *P*aO_2_. As this was associated with a reduced response of cerebral blood volume to hypocapnia, adaptation of cerebrovascular reactivity might have occurred.

## 1 Introduction

Approximately 140 million people live permanently at altitudes above 2,500 m (Moore et al., 1998). Due to a general growth in world-wide population, the number of people living at such altitudes can also assumed to be growing. Chronic altitude exposure and associated chronic hypoxaemia can have severe pathophysiological effects on the human body leading to chronic altitude related illnesses, such as high-altitude pulmonary hypertension (HAPH) (León-Velarde et al., 2005; Furian et al., 2015). HAPH is associated with excessive pulmonary vasoconstriction, further hypoxaemia, right heart dysfunction and potentially, premature death (León-Velarde et al., 2005). However, whether chronic hypoxaemia due to high-altitude residency or HAPH has an impact on cerebrovascular homeostasis and reactivity, has not been extensively studied.

Oxygen is essential for the brain; not only for its cognitive functions, but also to enable the brain to maintain its governor functions regulating numerous physiologic and metabolic processes. To ensure adequate oxygen delivery, cerebral blood vessels underlie calibre modulating autoregulatory mechanisms. They are activated by triggers such as fluctuations in systemic blood pressure (Paulson et al., 1972) or alterations in the partial pressure of the blood gases (Lassen and Christensen, 1976). Dropping arterial partial pressure of carbon dioxide (*P*aCO_2_) and rising arterial partial pressure of oxygen (*P*aO_2_), induce cerebral vasoconstriction, while hypercapnia and hypoxia induce cerebral vasodilation (Poulin et al., 2002; Ainslie and Burgess, 2008; Willie et al., 2014).

In 2012, we assessed cerebral tissue oxygenation (CTO), and shift in cerebral haemoglobin concentration (cHb) at baseline and in response to hypocapnia and hyperoxia, as an index of regional cerebral blood volume and cerebrovascular reactivity, in Kyrgyz highlanders and lowlanders at their respective altitude of residence (3,250 and 760 m), using non-invasive near-infrared spectroscopy (NIRS) (Imray et al., 1998; Al-Rawi et al., 2001; McLeod et al., 2003; Ulrich et al., 2014). To stimulate a cerebrovascular response, hyperoxia and hypocapnia, as well as their combination, were experimentally induced (Furian et al., 2015). We found similar CTO and cerebrovascular reactivity in highlanders (with and without HAPH) compared to a control group of healthy lowlanders, while arterial oxygen saturation (SpO_2_) at rest was significantly lower in highlanders. However, the cross-sectional study design did not allow assessment of possible observed compensation of CTO changes over time, especially in highlanders (Furian et al., 2015). Therefore, it remains unclear whether the similar CTO and cerebrovascular reactivity observed in highlanders and lowlanders are unbiased or due to some sort of natural selection or other causes.

Therefore, the purpose of the current longitudinal study was to repeat the identical measurements in these highlanders after 5 years of permanent high-altitude residency and investigate the progression of cerebral homeostasis and cerebrovascular reactivity in response to years living at high altitude and potential alterations in blood gases.

Based on the initial findings in 2012, we tested the hypotheses that in highlanders, CTO at rest remains preserved and increases under hyperoxia, while cHb decreases, as vasoconstriction occurs. Hypocapnia would reduce CTO and cHb. The combination of hyperoxia and hypocapnia presumably would cause cHb to decrease while CTO remains unaltered. Additionally, in a subgroup analysis, we investigated whether highlanders with and without HAPH differ in the progression of CTO and cerebral reactivity during the time course of 5 years.

## 2 Materials and methods

To assure comparability, the experimental design and measurement methods of the current study were identical to those of the previously published study in 2012 (Furian et al., 2015).

### 2.1 Participants

In 2012, Kyrgyz highlanders, born, raised, and currently living between 2,500 and 3,500 m, aged 18–80 years, were recruited on the Aksay high altitude plateau, and studied at the Aksay health post (3,250 m; barometric pressure 519 mmHg), Kyrgyzstan. The diagnosis of HAPH in 2012 was based upon a mean pulmonary artery pressure (mPAP) > 30 mmHg in the absence of excessive erythrocytosis (haemoglobin concentration in females <19 g/dL, in males <21 g/dL) and other diseases leading to hypoxaemia, such as cardiopulmonary diseases (León-Velarde et al., 2005). Exclusion criteria were other relevant cardiopulmonary disease, heavy smoking (>10 cigarettes per day or >25 pack-years) (Galie et al., 2016).

All participating highlanders from 2012 were invited to join the current study conducted in 2017 at the Aksay health post. The same exclusion criteria were applied. In conformity with a per-protocol analysis only data of subjects participating in both studies were analysed. Informed written consent was obtained, as well as approval by the ethics committee of the National Centre of Cardiology and Internal Medicine, Bishkek, Kyrgyzstan (01-8/433).

### 2.2 Design and interventions

Examination of the highlanders took place in supine position, breathing through a reservoir bag equipped face mask with a one-way exhalation valve. Ambient air was administered by a continuous positive airway pressure generator (REMstar; Philips Respironics, Zofingen, Switzerland). The fractional inspired oxygen (FiO_2_) mixtures of 100% (FiO_2_ 1.0), i.e., oxygen, and 21% (FiO_2_ 0.21), i.e., air, at a flow rate 10 L/min, were administered according to a randomized cross-over design. After a 20 min baseline period, a 20 min recording period followed, in which the participants were breathing air or oxygen, respectively. The participants were blinded to the inhaled gas mixture. At the end of each period, the participants were asked to hyperventilate until end-tidal partial pressure of carbon dioxide (PetCO_2_) was reduced by >10 mmHg for at least 15 s. Another period of 10 min of quiet breathing was affiliated, allowing the effects of hyperventilation, and if given hyperoxia, to wash out. Finally, the hyperventilation procedure was repeated with the alternative gas mixture. During the examination, the participants were not allowed to sleep.

### 2.3 Measurements

NIRS was performed using a NIRO 200NX device (Hamamatsu, Japan) emitting light at 735, 810 and 850 nm. Optode positioning in both studies followed the same protocol of bilateral placement at Fp1 and Fp2, high on the forehead, 4 cm apart, and secured with adhesive tape (Ulrich et al., 2014). The Hamamatsu NIRO device was chosen because of its use in our previous cross-sectional study performed on these highlanders. Its ability to track changes in CTO adequately when compared to the invasive techniques of jugular venous saturation and direct tissue probe measurement of brain oxygen tension is well established (Al-Rawi et al., 2001; McLeod et al., 2003). The NIRO 200NX device measures the changes in concentrations of oxygenated haemoglobin, ∆O_2_Hb (µmol/L), and deoxygenated haemoglobin ∆HHb (µmol/L) as well as the tissue oxygenation index (%), representing CTO. Changes in cHb (a surrogate for changes in blood volume) were calculated as ∆O_2_Hb + ∆HHb. NIRS data were recorded at 2 Hz along with ECG, finger pulse oximetry and capnography of expired air (Capnocheck Sleep; Smiths Medical PM Inc., Waukesha, WI, United States). The final 2 min of stable quiet breathing with FiO_2_ 0.21 and FiO_2_ 1.0 respectively, as well as the final 20–30 s of the hyperventilation plateau, were analysed. The data of the two NIRS channels were averaged. Arterial blood gases and haemoglobin concentration were obtained via a radial artery blood sample, drawn during quiet breathing with air (RapidPoint 405 & 500; Siemens, Zurich, Switzerland). Spirometry (EasyOne; NDD, Zurich, Switzerland) was performed with each participant (Miller et al., 2005) and Doppler echocardiography (SonoSite MicroMaxx; SonoSite Inc., Bothell, WA, United States) was used to measure the acceleration of peak velocity of pulmonary artery outflow, in order to derive mPAP (Kitabatake et al., 1983).

### 2.4 Statistics

All data were analysed using linear mixed regression models. Model fitting was tested with fitted residuals and random intercept QQ-plots. Due to the affirmed robustness of mixed models to violations of distributional assumptions, the normality of the data was considered irrelevant in the given data set (Arnau et al., 2013; Arnau et al., 2014; Schielzeth et al., 2020). In the mixed linear regression models, outcomes of interest were defined as dependent variable, whereas year of study (2017 vs. 2012) and intervention (air–rest, air–hyperventilation, oxygen–rest, oxygen–hyperventilation) were defined as fixed effects and participants as random effect. Data are presented as means ± SE. Differences among the tested conditions are expressed as mean difference with 95% confidence intervals. Further subgroup analysis was performed to evaluate variations taking the diagnosis of HAPH in 2012 into account. A probability of *p* < 0.05 was considered statistically significant. All statistical analyses were performed with R Studio (version 1.3.1056, R Studio Inc., San Francisco, United States) using the lmerTest package (version 3.1-2).

## 3 Results

**FIGURE 1:**
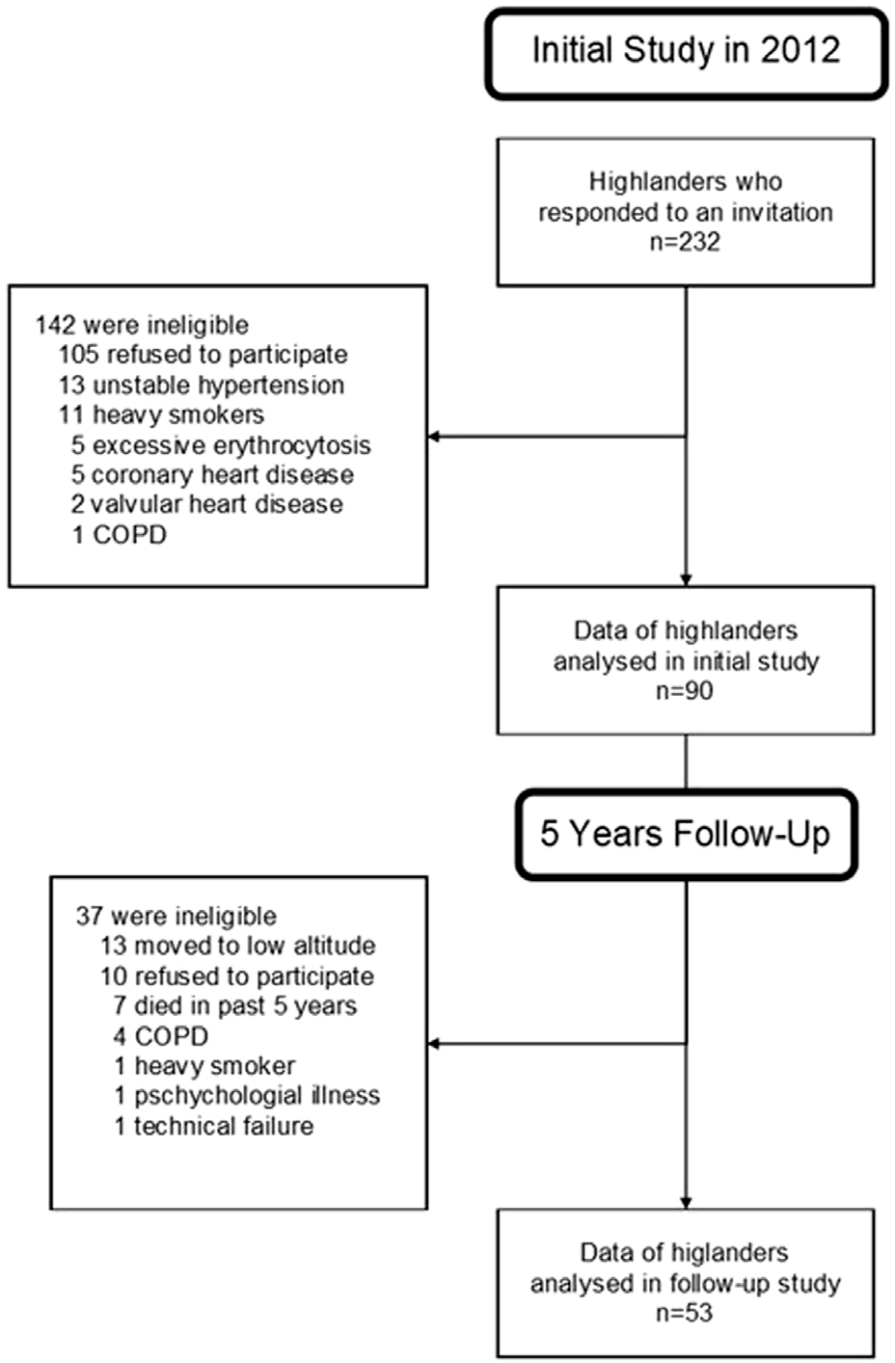
Study flow chart. Abbreviation: COPD, chronic obstructive pulmonary disease.

Of 90 highlanders who participated in 2012, 13 moved to low altitude, 10 refused to participate, 7 had died in the past 5 years, 6 were excluded due to the onset of new co-morbidities and 1 due to technical failure, resulting in 53 highlanders included in the final analysis (Figure 1).

**FIGURE 2:**
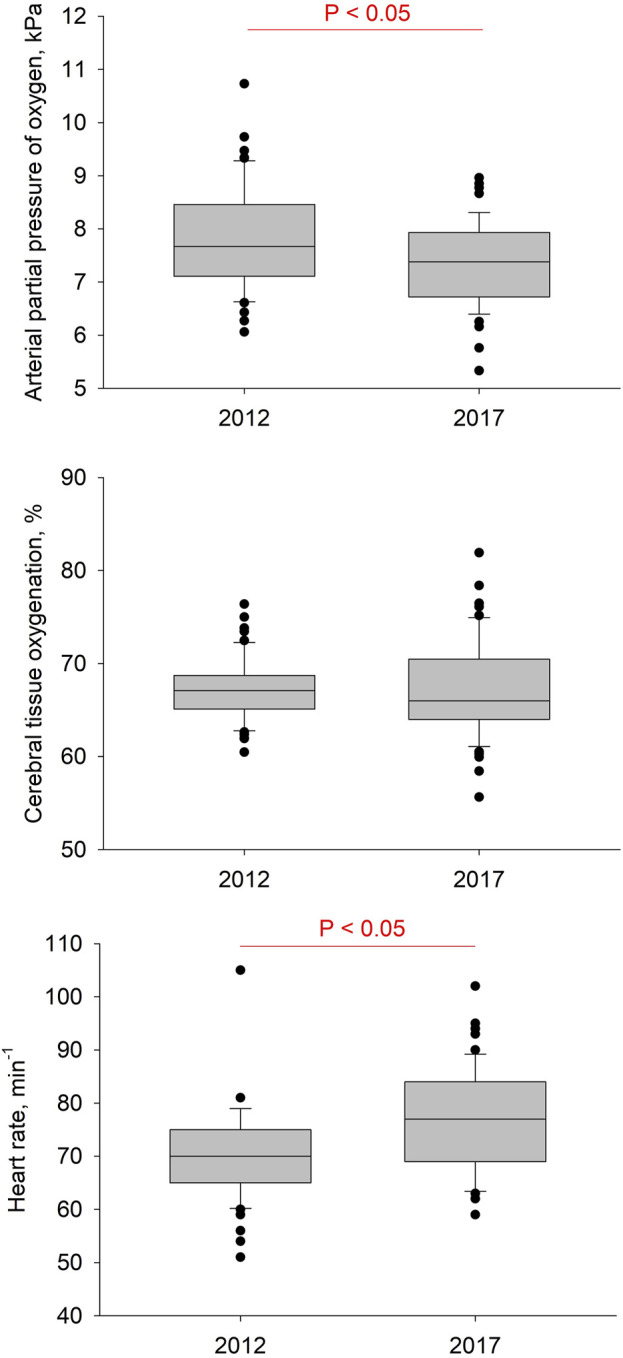
Results of baseline measurements at rest with ambient air. Boxes with lines represent medians and quartiles, whiskers represent the 10th and 90th percentiles, and dots represent individual values that fall outside the 10th–90th percentile range. Significant differences between years are indicated.

**FIGURE 3:**
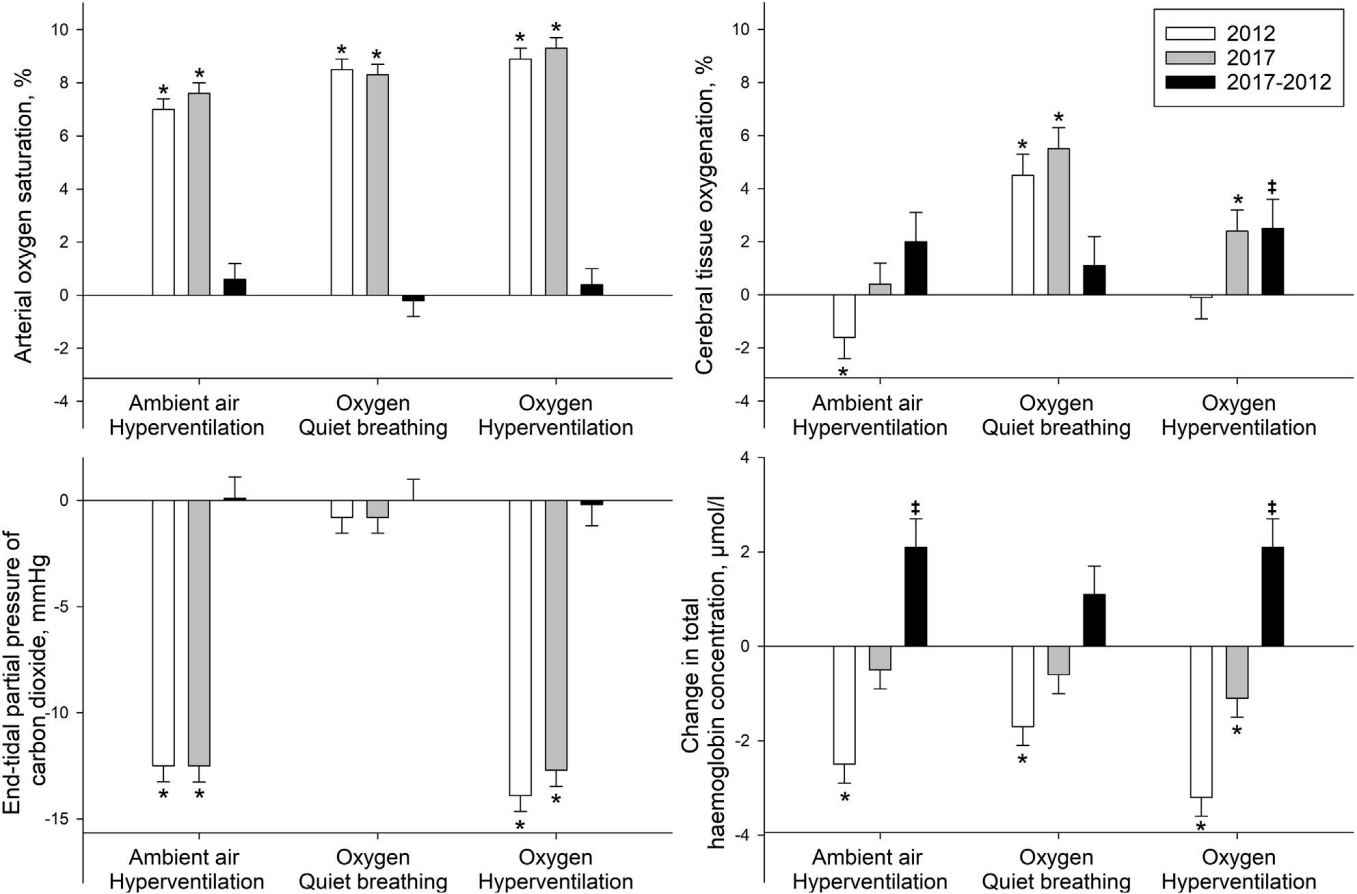
Change in physiological variables in response to hyperventilation and hyperoxia. Length of the bars show the given mean difference, whiskers show standard error, in comparison to quiet breathing under ambient air conditions (zero line, FiO2 0.21). *p < 0.05 significant difference versus breathing room air in same year; and ^‡^ *p* < 0.05 significant difference between the differences within the same intervention in 2017 compared to 2012.

Data for all baseline measurements are summarized in Table 1 and Figure 2. Measurements during the interventions are summarized in Table 2, Table 3 and Figure 3.

**TABLE 1:** Characteristics of study participants.

	2012	2017
N (% females)	53 (51)	53 (51)
Age, years	45 ± 2	50 ± 2*
Body mass index, kg/m^2^	25.8 ± 0.7	27.2 ± 0.7
Heart rate, min^−1^	70 ± 1	77 ± 1*
Systolic blood pressure, mmHg	113 ± 2	113 ± 2
Diastolic blood pressure, mmHg	74 ± 18	73 ± 1
Mean pulmonary arterial pressure, mmHg	27 ± 1	30 ± 1*
Arterial blood gases		
pH	7.43 ± 0.00	7.42 ± 0.00
*P*aO_2_, kPa	7.84 ± 0.13	7.40 ± 0.13*
*S*aO_2_, %	87.4 ± 0.6	86.2 ± 0.7
*P*aCO_2_, kPa	4.43 ± 0.07	4.44 ± 0.07
Hb concentration, g/dL	16.0 ± 0.3	16.3 ± 0.3

Values are means ± SE., *P*aO_2_, arterial partial pressure of oxygen; *S*aO_2_, arterial saturation of oxygen; *P*aCO2, arterial partial pressure of carbon dioxide; and Hb, haemoglobin.

*p < 0.05 versus corresponding value in 2012.

**TABLE 2:** Effects of hyperoxia and hypocapnia.

	Quiet breathing				Hyperventilation				
	FiO_2_ 0.21		FiO_2_ 1.0		FiO_2_ 0.21			FiO_2_ 1.0	
	2012	2017	2012	2017	2012		2017	2012	2017
SpO_2_, %	89.8 ± 0.4	89.0 ± 0.4	98.2 ± 0.4*	97.3 ± 0.4*^,‡^	96.7 ± 0.4*		96.7 ± 0.4*	98.7 ± 0.4*	98.4 ± 0.4*
CTO, %	67.2 ± 0.7	67.4 ± 0.7	71.7 ± 0.7*	72.9 ± 0.7*	65.6 ± 0.7*		67.8 ± 0.7^‡^	67.1 ± 0.7	69.8 ± 0.7*^,‡^
*P*etCO_2_, mmHg	32.8 ± 0.6	33.2 ± 0.6	31.9 ± 0.6	32.4 ± 0.6	20.2 ± 0.6*		20.7 ± 0.6*	20.2 ± 0.6*	20.5 ± 0.6*
HR, min^-1^	70 ± 1	77 ± 1^‡^	63 ± 1*	70 ± 1*^,‡^	68 ± 1		78 ± 1^‡^	67 ± 1*	76 ± 1^‡^
Bf, min^-1^	18 ± 1	20 ± 1^‡^	18 ± 1	20 ± 1^‡^	16 ± 1*		25 ± 1*^,‡^	15 ± 1*	24 ± 1*^,‡^

Values are means ± SE., FiO_2_, fractional inspired oxygen; SpO_2_, arterial oxygen saturation; CTO, cerebral tissue oxygenation; *P*etCO_2_, end-tidal partial pressure of carbon dioxide; HR, heart rate; and Bf, breathing frequency.

*p < 0.05 versus breathing room air breathing FiO_2_ 0.21 in the same year.

^‡^ *p* < 0.05 versus corresponding value in 2012.

**TABLE 3:** Response in cerebral tissue oxygenation and cerebrovascular reactivity to induced hypocapnia and hyperoxia.

	2012	2017	Difference between changes 2017 vs. 2012 *p*-value	
Rest, FiO_2_ 1.0 vs. FiO_2_ 0.21				
∆SpO_2_, %	8.5 (7.7–9.3)*	8.3 (7.5–9.1)*	−0.2 (−1.3 to 1.0)	0.772
∆CTO, %	4.5 (3.0–6.0)*	5.5 (4.1–7.0)*	1.1 (−1.0 to 3.1)	0.323
∆*P*etCO_2_, mmHg	−0.8 (−2.2 to 0.6)	−0.8 (−2.2 to 0.6)	0.0 (−1.9 to 1.9)	1.000
cHb, µmol/L	−1.7 (−2.9 to −0.6)*	−0.6 (−1.7 to 0.6)	1.1 (−0.5 to 2.7)	0.167
FiO_2_ 0.21, hyperventilation vs. rest				
∆SpO_2_, %	7.0 (6.1–7.8)*	7.6 (6.8–8.5)*	0.6 (−0.5–1.8)	0.284
∆CTO, %	−1.6 (−3.1 to −0.1)*	0.4 (−1.1–1.9)	2.0 (−0.1 to 4.1)	0.065
∆*P*etCO_2_, mmHg	−12.5 (−13.9 to −11.2)*	−12.5 (−13.8 to −11.1)*	0.1 (−1.8 to 2.0)	0.925
cHb, µmol/L	−2.5 (−3.7 to −1.4)*	−0.6 (−1.8 to 0.6)	2.0 (0.3–3.6)^‡^	0.019
FiO_2_ 1.0 hyperventilation vs. FiO_2_ 0.21 rest				
∆SpO_2_, %	8.9 (8.1–9.8)*	9.3 (8.5–10.2)*	0.4 (−0.8 to 1.6)	0.501
∆CTO, %	−0.1 (−1.6 to 1.4)	2.4 (0.9–3.9)*	2.5 (0.4–4.6)^‡^	0.020
∆*P*etCO_2_, mmHg	−12.5 (−13.9 to −11.2)*	−12.7 (−14.1 to −11.3)*	−0.2 (−2.1 to 1.8)	0.856
cHb, µmol/L	−3.2 (−4.3 to −2.0)*	−1.1 (−2.3 to 0.1)	2.1 (0.5–3.7)^‡^	0.012

Values are mean differences (95% confidence interval). FiO_2_, fractional inspired oxygen; SpO_2_, arterial oxygen saturation; CTO, cerebral tissue oxygenation; *P*etCO_2_, end-tidal partial pressure of carbon dioxide; and cHb, change in total haemoglobin concentration, a surrogate for cerebral blood volume, compared with quiet breathing at FiO_2_ 0.21.

*p < 0.05 significant difference vs. quiet breathing FiO_2_ 0.21 in the same year.

^‡^ *p* < 0.05 significant difference between the changes with the corresponding intervention in 2012 vs. 2017.

While breathing ambient air in 2017 compared to 2012, SpO_2_, CTO, *P*etCO_2_, *P*aCO_2_ and pH did not significantly differ, whereas *P*aO_2_ significantly decreased. Blood pressure did not significantly change, while mPAP, heart rate and breathing frequency rose significantly.

Breathing oxygen increased CTO and SpO_2_ similarly in 2017 and 2012. *P*etCO_2_ did not show significant changes neither compared to air, between 2017 and 2012. cHb decreased significantly in 2012 compared to breathing air, whereas it remained unchanged in 2017 (Table 3).

Hyperventilation while breathing ambient air decreased CTO significantly in 2012 while it slightly but insignificantly rose in 2017. The significant rise in SpO_2_ did not change over 5 years, as well as the reduction of *P*etCO_2_ by >10 mmHg did not change. cHb decreased significantly in 2012 but remained stable in 2017 with a significant difference between the years (Table 3).

Hyperventilation while breathing oxygen had no impact on CTO in 2012 but showed a significant increase in 2017 and also a significant difference between years. The significant rise in SpO_2_ did not change over the 5 years, nor did the reduction of *P*etCO_2_ by >10 mmHg did not change, however, like hyperventilation while breathing ambient air, cHb did not decrease as seen in 2012.

The interventions revealed that the overall increase in CTO during hyperventilation (FiO_2_ 1.0) in 2017 vs. 2012 was mainly attributable to the HAPH group (71.7% ± 1.2% vs. 67.9% ± 1.2% HAPH; 68.6% ± 0.9% vs. 66.6% ± 0.9% highlanders without initially diagnosed HAPH) (data not shown), whereas the reduction in cHb during the same interventions followed a similar pattern in both groups (Table 5). The most evident difference in both groups was the uniformly higher SpO_2_ value in the highlanders without initially diagnosed HAPH group throughout all measurements.

## 4 Discussion

This prospective cohort study in highlanders permanently living above 2,500 m showed that after 5 years at high altitude we observe a reduction in *P*aO_2_ and increased HR and mPAP (Table 1) while CTO (FiO_2_ 0.21 during quiet breathing) remained preserved (Figure 2). However, after 5 years, cerebrovascular reactivity in response to hypocapnia and hyperoxia revealed less stringent cerebrovascular vasoconstriction, which resulted in an increase of CTO compared to the same interventions in 2012. Whether these observed alterations in the cerebrovascular responsiveness should be interpreted as beneficial, due to maintenance of CTO, or deleterious, due to increased vascular stiffness and therefore less vasoconstriction, remains to be elucidated.

Known triggers of cerebral vasoconstriction, namely, hyperoxia and hypocapnia as well as their combination, were experimentally induced (Tables 2, 3; Figure 3). Hypocapnia was induced by hyperventilation and hyperoxia by breathing oxygen. Hyperventilation while breathing ambient air decreased CTO in 2012, but CTO remained unaffected in 2017. Hyperoxia increased CTO in both studies significantly. In 2017, we saw a trend for an even greater increase. The combination of hyperoxia and hyperventilation had little or no impact on CTO in 2012, but significantly increased CTO in 2017. cHb, which indicates changes of regional cerebral blood volume, displayed similar alterations in all three interventions. In 2012, it was significantly reduced, whereas in 2017, it remained unchanged or was reduced to a much lesser extent. Therefore, all changes reflecting cerebrovascular reactivity in response to the applied interventions indicate a mitigated response of cerebral blood vessels to the tested stimuli after 5 years living at high altitude. These results confirm findings from our previous cross-sectional study performed on these highlanders. Furian et al. (2015) reported preserved CTO in highlanders compared to healthy lowlanders at their respective altitude during resting conditions. However, during hypocapnia CTO in highlanders was essentially preserved, unlike the CTO reductions observed in healthy lowlanders. These observations are in accordance with the suggestion, that altitude-related adaptations of the cerebrovascular reactivity took place in these highlanders over the course of 5 years.

To gain further insight on whether these alterations in cerebrovascular reactivity were related to the presence of HAPH, exploratory subgroup analysis in highlanders diagnosed with HAPH in 2012 compared to highlanders without HAPH was performed. It revealed a similar pattern of reduced cerebrovascular reactivity in highlanders with and without HAPH. Therefore, the observed cerebrovascular changes were promoted by chronic hypoxaemia and age, rather than by differences in haemodynamic (Table 4).

**TABLE 4:** Effects of hyperoxia and hypocapnia of subgroups.

	Quiet breathing				Hyperventilation			
	FiO_2_ 0.21		FiO_2_ 1.0		FiO_2_ 0.21		FiO_2_ 1.0	
	2012	2017	2012	2017	2012	2017	2012	2017
In highlanders with HAPH								
SpO_2_, %	87.1 ± 0.6	86.7 ± 0.6	97.6 ± 0.6*	96.2 ± 0.6*^,‡^	95.9 ± 0.6*	95.8 ± 0.6*	98.4 ± 0.6*	97.8 ± 0.6*
CTO, %	67.0 ± 1.2	68.3 ± 1.2	72.5 ± 1.2*	74.6 ± 1.2*	66.5 ± 1.2	69.4 ± 1.2^‡^	67.9 ± 1.2	71.7 ± 1.2*^,‡^
*P*etCO_2_, mmHg	33 ± 1	34 ± 1	32 ± 1	33 ± 1	21 ± 1*	22 ± 1*	20 ± 1*	22 ± 1*
HR, min^-1^	75 ± 2.1	80 ± 2.1^‡^	68 ± 2.1*	73 ± 2.1*^,‡^	73 ± 2.1	79 ± 2.1^‡^	71 ± 2.1*	79 ± 2.1^‡^
Bf, min^-1^	19 ± 0.8	21 ± 0.8	18 ± 0.8	20 ± 0.8	15 ± 0.8*	25 ± 0.8*^,‡^	15 ± 0.8*	24 ± 0.8*^,‡^
In highlanders without HAPH in 2012								
SpO_2_, %	91.4 ± 0.4^†^	90.4 ± 0.4^†^	98.6 ± 0.4*	98.0 ± 0.4*^,†^	97.2 ± 0.4*	97.2 ± 0.4*^,†^	98.9 ± 0.4*	98.7 ± 0.4*
CTO, %	67.3 ± 0.9	66.8 ± 0.9	71.2 ± 0.9*	71.9 ± 0.9*	65.0 ± 0.9*	66.7 ± 0.9	66.6 ± 0.9	68.6 ± 0.9^†,‡^
*P*etCO_2_, mmHg	33 ± 1	33 ± 1	32 ± 1	32 ± 1	20 ± 1*	20 ± 1*^,†^	20 ± 1*	20 ± 1*
HR, min^−1^	67 ± 1.7†	75 ± 1.7‡	60 ± 1.7*^,†^	68 ± 1.7*‡	66 ± 1.7^†^	77 ± 1.7^‡^	64 ± 1.7^†^	75 ± 1.7^‡^
Bf, min^−1^	18 ± 0.7	20 ± 0.7	17 ± 0.7	19 ± 0.7‡	16 ± 0.7*	24 ± 0.7*^,‡^	15 ± 0.7*	24 ± 0.7*^,‡^

Values are means ± SE. CTO, cerebral tissue oxygenation; FiO_2_, fractional inspired oxygen; PetCO_2_, end-tidal partial pressure of carbon dioxide; SpO_2_, peripheral arterial oxygen saturation; HR, heart rate; and Bf, breathing frequency.

*p < 0.05 versus quiet breathing, FiO_2_ 0.21 in same group and year.

^†^ *p* < 0.05 versus highlanders with HAPH, in same year.

^‡^ *p* < 0.05 versus corresponding value in same group in 2012.

**TABLE 5:** Response in cerebral tissue oxygenation and cerebrovascular reactivity to induced hypocapnia and hyperoxia in subgroups.

	2012		2017		Difference between changes 2017 vs. 2012			*p*-value
Rest, FiO_2_ 1.0 vs. FiO_2_ 0.21								
In highlanders with HAPH								
∆SpO_2_, %	10.5 (9.2–11.8)*		9.5 (8.2–10.8)*		−1.0 (−2.8 to 0.8)			0.305
∆CTO, %	5.5 (3.1–7.9)*		6.3 (3.9–8.7)*		0.7 (−2.6 to 4.1)			0.671
∆*P*etCO_2_, mmHg	−1.3 (−3.5 to 1)		−0.9 (−3.1 to 1.4)		0.4 (−2.7 to 3.5)			0.804
cHb, µmol/L	−1.8 (−3.1 to −0.4)*		−0.6 (−2 to 0.7)		0 (−0.7 to 3)			0.992
In highlanders without HAPH in 2012								
∆SpO_2_, %	7.3 (6.3–8.3)*		7.6 (6.6–8.6)*		0.3 (−1.1 to 1.7)			0.677
∆CTO, %	3.9 (2–5.7)*		5.1 (3.2–7)*		1.2 (−1.4–3.9)			0.357
∆*P*etCO_2_, mmHg	−0.5 (−2.3 to 1.2)		−0.8 (−2.5 to 1)		−0.2 (−2.7 to 2.2)			0.847
cHb, µmol/L	−1.7 (−2.7 to −0.6)*		−0.5 (−1.6 to 0.5)		1.1 (−0.3 to 2.6)			0.134
FiO_2_ 0.21, hyperventilation vs. rest								
In highlanders with HAPH								
∆SpO_2_, %	8.8 (7.5–10.1)*		9.1 (7.8–10.4)*		0.3 (−1.5 to 0.8)			0.761
∆CTO, %	−0.5 (−2.9 to 1.9)		1.1 (−1.3 to 3.5)		1.6 (−1.8 to 4.1)			0.366
∆*P*etCO_2_, mmHg	−12.5 (−14.7 to −10.3)*		−11.7 (−13.9 to −9.4)*		0.9 (−2.3 to 3.5)			0.598
cHb, µmol/L	−2 (−3.3 to −0.6)*		−0.7 (−2.1 to 0.7)		0.1 (−0.6 to 3)			0.901
In highlanders without HAPH in 2012								
∆SpO_2_, %	5.9 (4.9–6.9)*		6.7 (5.7–7.8)*		0.9 (−0.6 to 2.3)			0.243
∆CTO, %	−2.3 (−4.2 to −0.4)*		−0.1 (−2 to 1.8)		2.2 (−0.4 to 4.8)			0.108
∆*P*etCO_2_, mmHg	−12.6 (−14.3 to −10.8)*		−13 (−14.8 to −11.2)*		−0.4 (−2.9 to 2)			0.745
cHb, µmol/L	−2.9 (−3.9 to −1.8)*		−0.3 (−1.4 to 0.8)		2.6 (1.1–4.1)‡			0.001
FiO_2_ 1.0, hyperventilation vs. FiO_2_ 0.21, rest								
In highlanders with HAPH								
∆SpO_2_, %	11.3 (10–12.5)*		11.1 (9.8–12.4)*		−0.1 (−1.9 to 1.7)			0.885
∆CTO, %	0.9 (−1.5–3.4)		3.4 (1–5.8)*		2.4 (−0.9 to 5.8)			0.163
∆*P*etCO_2_, mmHg	−12.7 (−14.9 to −10.5)*		−12.4 (−14.6 to −10.2)*		0.3 (−2.8 to 3.4)			0.852
cHb, µmol/L	−3.6 (−5 to −2.3)*		−1.2 (−2.5 to 0.2)		1.3 (0.6–4.3)			0.277
In highlanders without HAPH in 2012								
∆SpO_2_, %	7.5 (6.5–8.6)*		8.3 (7.2–9.3)*		0.7 (−0.7 to 2.1)			0.340
∆CTO, %	−0.7 (−2.6 to 1.1)		1.8 (−0.2 to 3.7)		2.5 (−0.1 to 5.1)			0.069
∆*P*etCO_2_, mmHg	−12.4 (−14.2 to −10.7)*		−12.9 (−14.7 to −11.1)*		−0.5 (−3 to 1.9)			0.687
cHb, µmol/L	−2.9 (−4 to −1.9)*		−1.1 (−2.2 to 0.0)*		1.8 (0.4–3.3)‡			0.016
HAPH vs. highlanders without HAPH in 2012, differences between 2017 vs. 2012								
	Int1	*p*-value	Int2	*p*-value		Int3	*p*-value	
∆SpO_2_, %	0.6 (−1.7–2.9)	0.629	1.3 (−1–3.5)	0.287		0.8 (−1.5–3.1)	0.481	
∆CTO, %	0.6 (−3.6–4.9)	0.776	0.5 (−3.7–4.7)	0.818		0.1 (−4.2–4.3)	0.973	
∆*P*etCO_2_, mmHg	−1.3 (−5.2 to 2.7)	0.538	−0.6 (−4.6 to 3.3)	0.753		−0.8 (−4.8 to 3.1)	0.693	
cHb, µmol/L	1.5 (−1–3.7)	0.214	0 (−2.4 to 2.3)	0.979		−1.9 (−3 to 1.8)	0.271	

Values are mean differences (95% confidence interval). SpO_2_, peripheral arterial oxygen saturation; CTO, cerebral tissue oxygenation; FiO_2_, fractional inspired oxygen; *P*etCO_2_, end-tidal partial pressure of carbon dioxide; cHb, change in total haemoglobin concentration, a surrogate for cerebral blood volume, compared with quiet breathing at FiO2 0.21.

*p < 0.05 significant difference versus quiet breathing, FiO_2_ 0.21 under rest in same year.

^‡^ *p* < 0.05 significant difference between the differences 2012 vs. 2017 in same group.

When elaborating the effect of chronic hypoxaemia, we saw worsening of arterial oxygenation over 5 years, therefore, a hypocapnic manoeuvre results in a higher increase in arterial oxygenation vs. decreased CO_2_. This balance towards higher elevation in SpO_2_ with hypocapnia would induce a larger increase in CTO compared to the induced cerebral vasoconstriction. This agrees with previous findings by Furian et al. (2015). Another mechanism might be progression of hypoxaemia-related arterial blood vessel stiffness resulting in lower blood vessel reactivity in response to hypocapnia and hypoxaemia. This might have promoted less vasoconstriction and therefore, higher CTO values in response to better oxygenated arterial blood under hyperventilation. It remains to be elucidated whether the potential blood vessel stiffness was induced by aging (Kucharska-Newton et al., 2019) or hypoxaemia. Further, the elevated pulmonary artery pressure in highlanders may compromise venous drainage in the brain, causing increased cerebral blood volume, which may additionally interfere with the regulation of cerebral blood flow (Ainslie and Ogoh, 2010).

This longitudinal cohort study was carried out on Kyrgyz highlanders living between 2,500 and 3,500 m. Whether the cerebrovascular adaptations to protect brain metabolism are preserved in highlanders living at higher altitudes or with different ethnic backgrounds remains to be confirmed. Further, since chronic hypoxaemia might have induced vasodilatation of the cerebral blood vessels, changes in cHb (a surrogate for blood volume) might not represent the same degree of cerebral vasoconstriction in 2017 compared to 2012. Additional measurements to assess the cerebrovascular reactivity (i.e., cold pressor test) would have provided further insights into the physiology and adaptation of highlanders to chronic hypoxia. However, due to the paired comparisons, highlanders served as their own control group, so this potential bias was minimized. Due to the collinear change in age, mPAP and hypoxaemia, the likely principal mechanism underlying the observed cerebrovascular changes needs further study (Lovallo, 1975).

This first longitudinal cohort study on a large number of highlanders revealed decreased arterial oxygenation but preserved CTO after 5 years of living at high altitude. CTO was preserved in contempt of blunted cerebrovascular reactivity in response to hypocapnia and hyperoxia. These findings were observed in both, highlanders with and without initially diagnosed HAPH, despite unique mPAP progression in highlanders without initially diagnosed HAPH and preserved mPAP in highlanders with HAPH. These findings suggest that the altered cerebrovascular reactivity might be caused by progression in cerebral vessel stiffness due to chronic hypoxaemia or aging, or the combination of both. Whether the observed alterations in the cerebrovascular reactivity are beneficial, by maintaining CTO, or deleterious, remains to be elucidated.

## Data Availability

The original contributions presented in the study are included in the article/Supplementary Material, further inquiries can be directed to the corresponding author.

## Abbreviations

Bf, Breathing frequency; cHb, Change in cerebral haemoglobin concentration; COPD, Chronic obstructive pulmonary disease; CTO, Cerebral tissue oxygenation; FiO_2,_ Fractional of inspired oxygen; HAPH, High-altitude pulmonary hypertension; HR, Heart rate; mPAP, Mean pulmonary artery pressure; NIRS, Near-infrared spectroscopy; *P*aCO_2_, Arterial partial pressure of carbon dioxide; *P*aO_2_, Arterial partial pressure of oxygen; *P*etCO_2_, End-tidal partial pressure of carbon dioxide; SaO_2_, Arterial oxygen saturation; SpO_2_, Saturation of oxygen.
